# Supplementary material for: Modulation of perovskite degradation with multiple-barrier for light-heat stable perovskite solar cells
Source: Nat Commun. 2023 Sep 30;14:6120. doi: 10.1038/s41467-023-41856-9 (PMC10542753; doi:10.1038/s41467-023-41856-9)
Supplement: Supplementary file 1 — Supplementary Information [file 41467_2023_41856_MOESM1_ESM.pdf]

Supplementary Information

for

Modulation of perovskite degradation with multiple-barrier for light-heat stable perovskite solar cells

Jing Zhou[†1], Zonghao Liu[†1,2*], Peng Yu[†1,3], Guoqing Tong[†4], Ruijun Chen[5], Luis K. Ono[4], Rui Chen[1], Haixin Wang[1], Fumeng Ren[1], Sanwan Liu[1], Jianan Wang[1], Zhigao Lan[5], Yabing Qi[4*] and Wei Chen[1,2*]

[1]Wuhan National Laboratory for Optoelectronics (WNLO), Huazhong University of Science and Technology (HUST), Wuhan 430074, China.

[2]Optics Valley Laboratory, Hubei, 430074 China.

[3]China-EU Institute for Clean and Renewable Energy, Huazhong University of Science and Technology, Wuhan 430074, Hubei, China.

[4]Energy Materials and Surface Sciences Unit (EMSSU), Okinawa Institutes of Science and Technology Graduate University (OIST), 1919-1 Tancha, Onna-son, Kunigami-gun, Okinawa 904-0495, Japan.

[5]School of Physics and Telecommunications, Huanggang Normal University, Huanggang 438000, Hubei, China.

† These authors contributed equally to this work.

*Corresponding authors. E-mail: liuzonghao@hust.edu.cn (Z. L.); Yabing.Qi@OIST.jp (Y. B. Q.); wnlochenwei@hust.edu.cn (W. C.).

Supplementary Note 1

To quantitively evaluate the barrier capability of the Al_2O_3/parylene bilayer, we tested the water and oxygen transmittance parameters (WVTR) of each layer and bilayer by weight method. The WVTRs of single Al_2O_3 film, single parylene film, and Al_2O_3/parylene bilayer are determined to be 2.24×10^{-4}, 1.38×10^{-4}, and 7.12×10^{-5} g m^{-2} day^{-1} respectively. This enhanced barrier capability of the Al_2O_3/parylene bilayer than the single layers is because the Al_2O_3 layer can prevent water and oxygen permeation, and parylene films decouple the defects in the Al_2O_3 inorganic layer.[1] The much lower WVTR of the Al_2O_3/parylene bilayer versus the single layers verifies the bilayer as a strong chemical permeation barrier. To further intuitively evaluate the protective effect of Al_2O_3/parylene bilayer on perovskite against water ingress, we immersed perovskite films with single layers, Al_2O_3/parylene bilayer and without barrier into water (Supplementary Figure 3 and Supplementary Movie 1). The results further verify that the Al_2O_3/parylene bilayer shows much better protective capability than the Al_2O_3 or parylene single layer.

Supplementary Note 2

To further explore the effect of encapsulation on the optoelectrical properties evolutions of perovskite films under constant high-temperature stress, we characterized the optical and corresponding 2D-PL mapping evolution of the FA-based perovskite films at 170 °C in N_2 atmosphere. Supplementary Figure 4a shows the images of perovskite films with or without encapsulation after 170 °C annealing for different time. It is observed that the FA-based perovskite film encapsulated with bilayer barrier retained black after 30 hours annealing, while the perovskite film without barrier or just single parylene layer turned to yellow within 5 hours and the film with only Al_2O_3 layer can retain its original color for a little bit longer time. These results suggest that ALD-Al_2O_3 film shows better blocking capability when compared with the CVD-parylene layer. This may be due to that the CVD based polymer film is usually with a relatively looser structure at molecular level, which is not as compact as ALD-Al_2O_3 film. Although ALD-Al_2O_3 film is more compact than parylene films, the single layer still can't completely retard the corrosion of water vapor in the air for a long time. When combined CVD-parylene with ALD-Al_2O_3 film, the hydrophobic parylene layer can impede condensation of water vapor on the Al_2O_3 film so as to achieve a more immaculate structure, which can realize a preferable protection on devices[2,3]. The corresponding 2D-PL mapping of the corresponding samples was also measured (Supplementary Figure 4b). As shown in the PL mapping images, the films with bilayer barrier maintained uniform and unchanged fluorescence intensities, which confirms the effective protective effect of the barriers on perovskite. The above results reveal that the Al_2O_3/parylene barrier can remarkably improve the thermal stability of the perovskite, which also suggests that the use of robust encapsulation can effectively modulate of perovskite degradation from the viewpoint of reaction thermodynamics.

Supplementary Note 3

To further examine the hydrothermal stability of the perovskite film with Al_2O_3/parylene bilayer, the samples were aged at 85 °C/85% relative humidity environment. As shown in Supplementary Figure 5a, the perovskite films without barrier and with a single parylene barrier have decomposed completely after 30 hours of aging. Partial region of the film with the Al_2O_3 single barrier was still brown, the degraded yellow region maybe due to the inferior compactness of the Al_2O_3 film with defects allowing the ingress of H_2O and outgassing of perovskite degradation products. Surprisingly, the film with Al_2O_3/parylene bilayer barrier still remained its original brown color, which further verifies its robust barrier capability. This is further supported by the microscopic morphology changes measured by scanning electron microscopy (SEM) characterization (Supplementary Figure 5b) and 2D-PL mapping measurements (Supplementary Figure 5c and Supplementary Figure 7a). We further investigated the influence of the above barriers on light-heat stability of perovskite under 1 sun equivalent white light LED illumination at 85 °C heating in ambient air (Supplementary Figure 6 and Supplementary Figure 7b). Consistent with the above aging test results, a single Al_2O_3 or a parylene barrier could improve the stability to a certain degree, and Al_2O_3/parylene bilayer-based sample shows the best stability.

Supplementary Note 4

The technical scalability of these thin-film barriers involved in our work is discussed as follows:

(i) Regarding the ALD deposition of Al_2O_3, spatial ALD deposition is a promising choice, since it has been widely applied in industry for the deposition of passivation layer within commercialized crystalline Si solar cell, which can be used to deposit tens of nanometers of metal oxide in the scale of 1 m × 2 m within several minutes;

(ii) CVD deposition of parylene has been widely employed for room-temperature electronic encapsulation in various fields. The large-scale machine for parylene deposition is also not expensive.

(iii) Magnetron sputtering is one of the most widely employed techniques for thin film deposition in industry. Here, magnetron sputtering is employed to deposit Bi electrode instead of thermal evaporation because of its better conductivity and morphological compactness. Besides, magnetron sputtering is also superior in increasing the electrode deposition rate and utilization rate of raw materials for scalable production when compared with thermal evaporation. Furthermore, due to the low melting point and higher vapor pressure of Bi under vacuum, it is found it is very easy to deposit Bi film safely on the top of perovskite. A relatively low deposition power is enough to achieve a relatively high deposition rate of Bi than Mo and ITO films.

Thus, these techniques could be easily integrated into the industrial production line of perovskite solar modules.

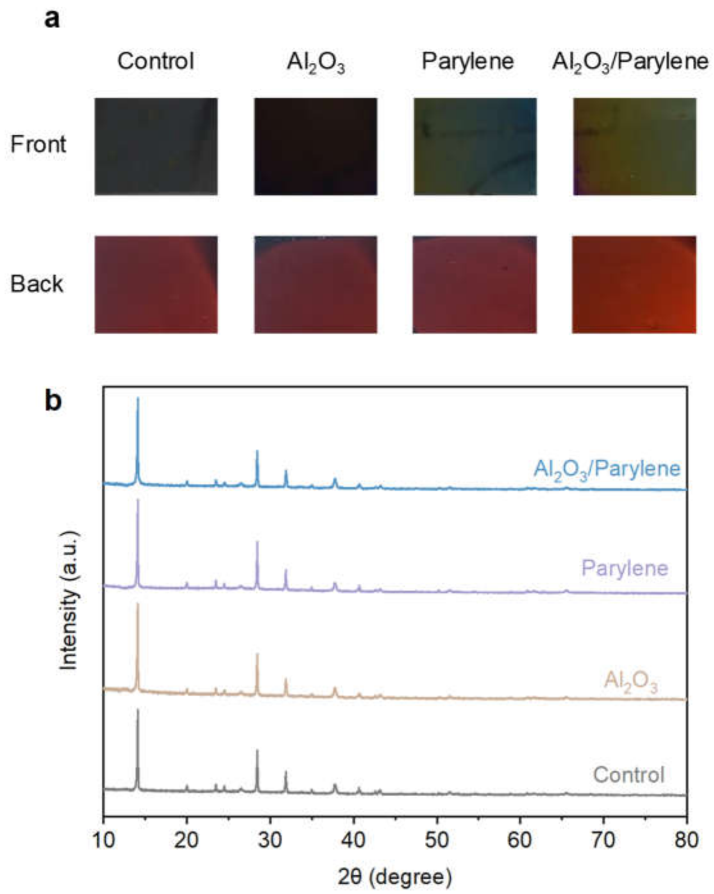

Supplementary Figure 1 | Changes in perovskite film before and after the deposition of the barrier. a, Photos of the perovskite samples with different barriers: no barrier, single Al_2O_3, single parylene and Al_2O_3/parylene bilayer. **b,** XRD spectra of the samples prepared by packaging different barriers: no barrier, single Al_2O_3, single parylene and Al_2O_3/parylene bilayer.

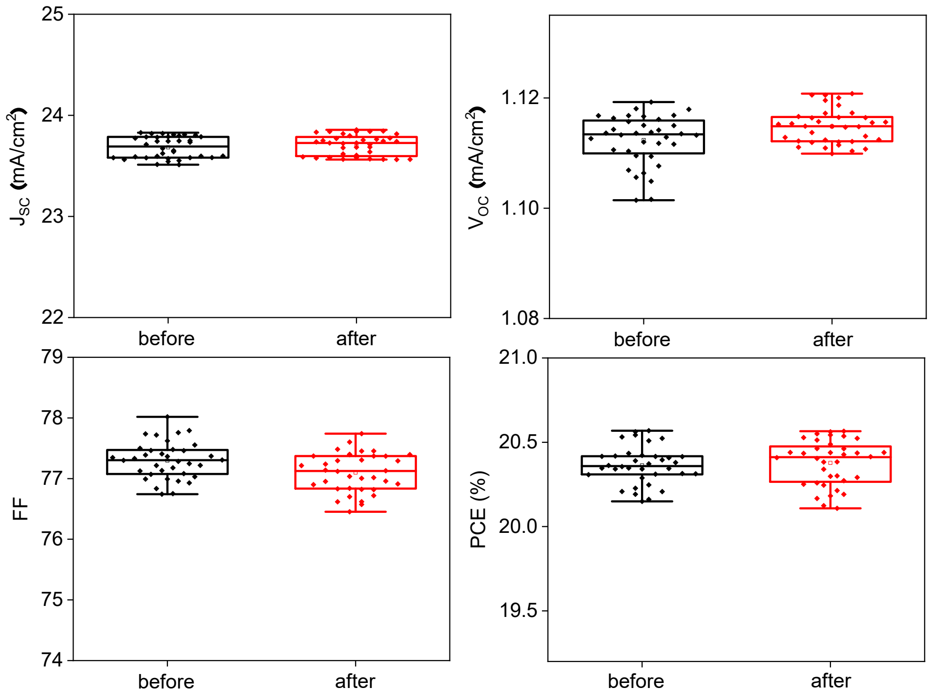

Supplementary Figure 2 | Impact of bilayer on the device performance of FA-PSCs (18 devices, forward and reverse scans for each device) with Bi electrode (1 cm^2) (before and after packaging with a bilayer).

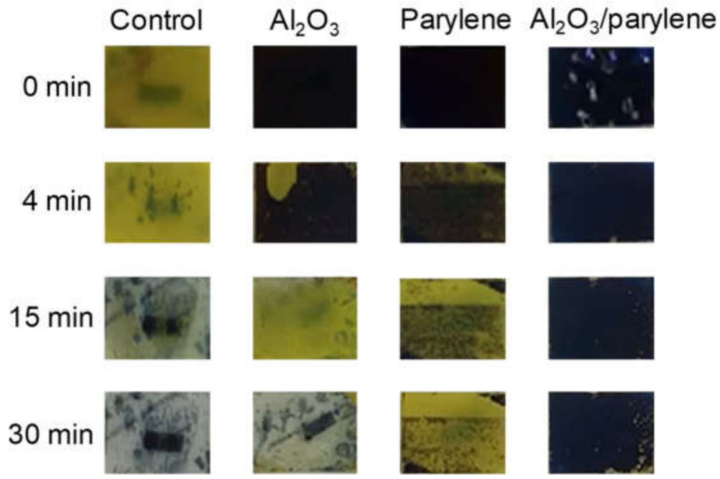

Supplementary Figure 3 | The optical image evolutions of the perovskite films with different encapsulation conditions during the water soaking test.

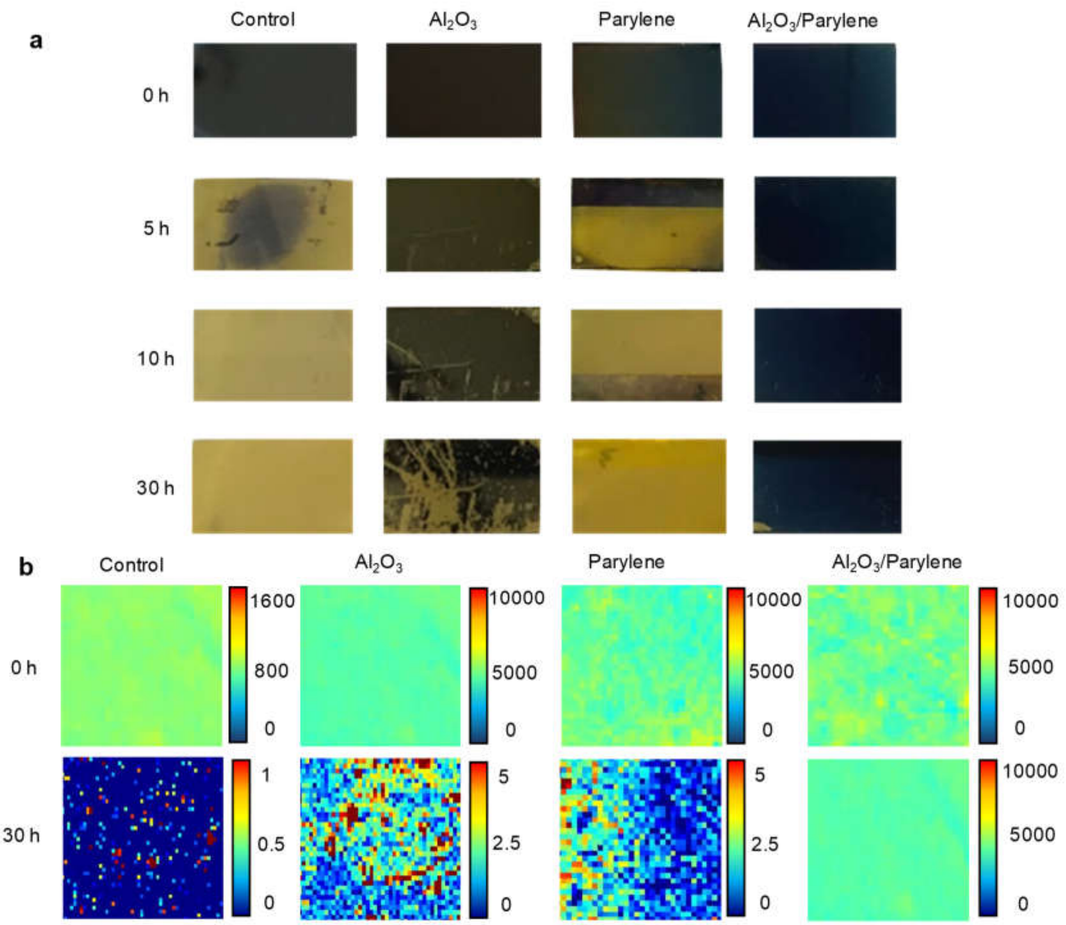

Supplementary Figure 4 | Thermal aging test of the FA-based perovskite films aging at 170 °C in N_2 atmosphere. a, Optical photographs for the changing process of the perovskite films with different encapsulation conditions, including the control film without barrier, the film with Al_2O_3 barrier, the film with parylene barrier, and the film with Al_2O_3/parylene bilayer barrier. **b,** 2D PL-Mapping of the FA-based perovskite films with different encapsulation conditions aging at 170 °C in N_2 atmosphere.

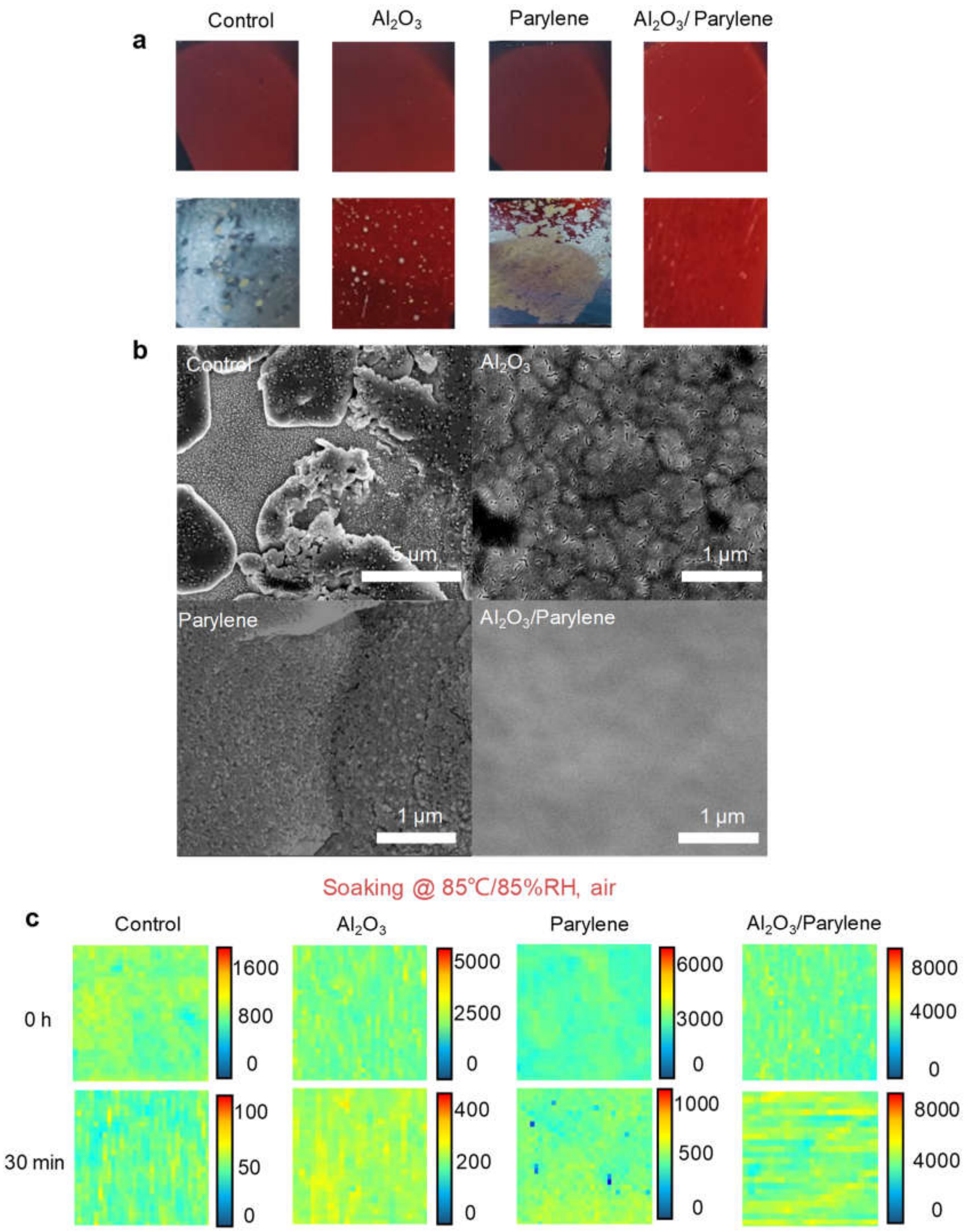

Supplementary Figure 5 | 85 °C/85%RH aging test of the FA-based perovskite films. a, optical photographs for the changing process of the perovskite film with different encapsulation conditions, including the control film without barrier, the film with Al_2O_3 barrier, the film with parylene barrier, and the film with Al_2O_3/parylene bilayer barrier. **b,** SEM images of the aged perovskite films with different encapsulation conditions (scare bar: 1 μm). **c,** 2D PL-Mapping of the perovskite films with different encapsulation conditions aging at 85 °C and 85% RH.

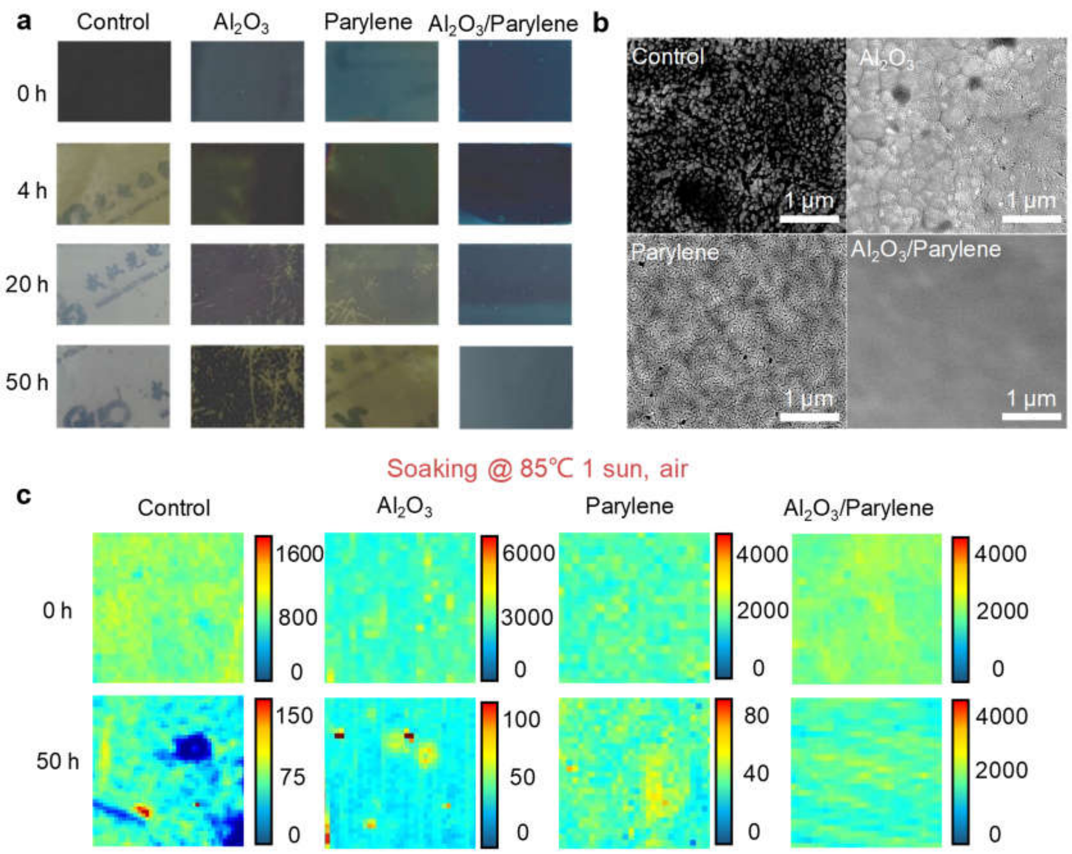

Supplementary Figure 6 | Light-heat aging test of the FA-based perovskite films with and without barriers, including the control film without barrier, the film with Al_2O_3 barrier, the film with parylene barrier, and the film with Al_2O_3/parylene barrier, under 1 sun equivalent continuous white light LED illumination with 85 ℃ heating. a, Optical photographs for the evolutions of the perovskite films with different thin-film barriers. **b,** SEM images of the aged perovskite films with different barriers (scare bar: 1 μm). **c,** 2D PL-Mapping of the perovskite films with different barriers aging at 85 °C soaking with 1 sun equivalent white light LED illumination.

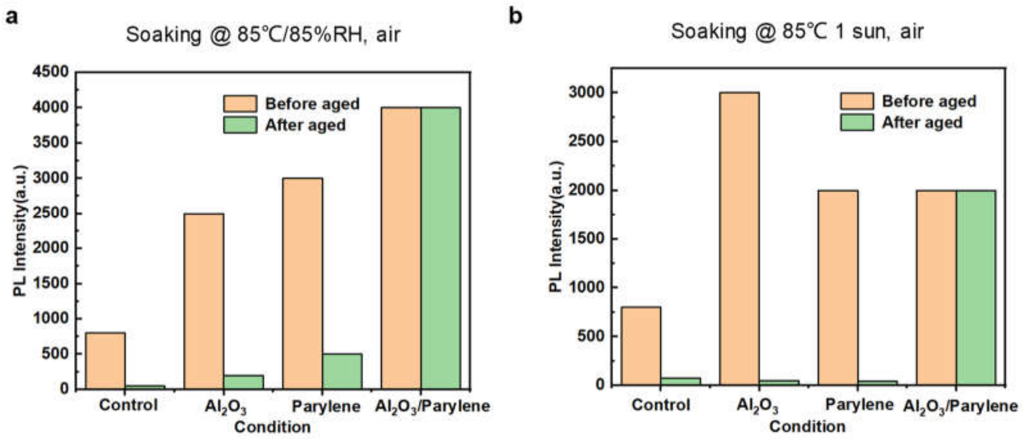

Supplementary Figure 7 | Fluorescence intensity variation of perovskite film under different aging conditions. a, Fluorescence intensity statistic of the perovskite films with different encapsulation conditions aging at 85 ℃ and 85% RH. **b,** Fluorescence intensity statistic of the perovskite films with different encapsulation conditions aging at 85 ℃ soaking in 1 sun equivalent white light LED illumination.

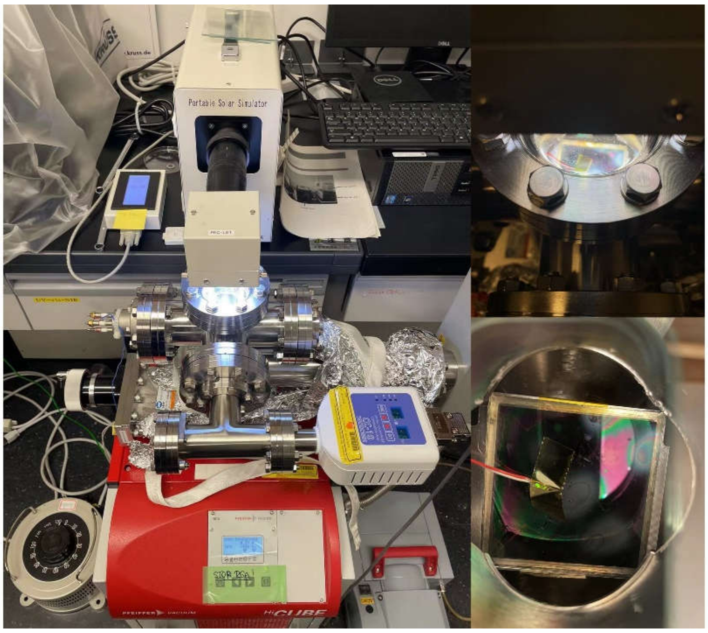

Supplementary Figure 8 | The equipment for in situ mass spectrometry measurements.

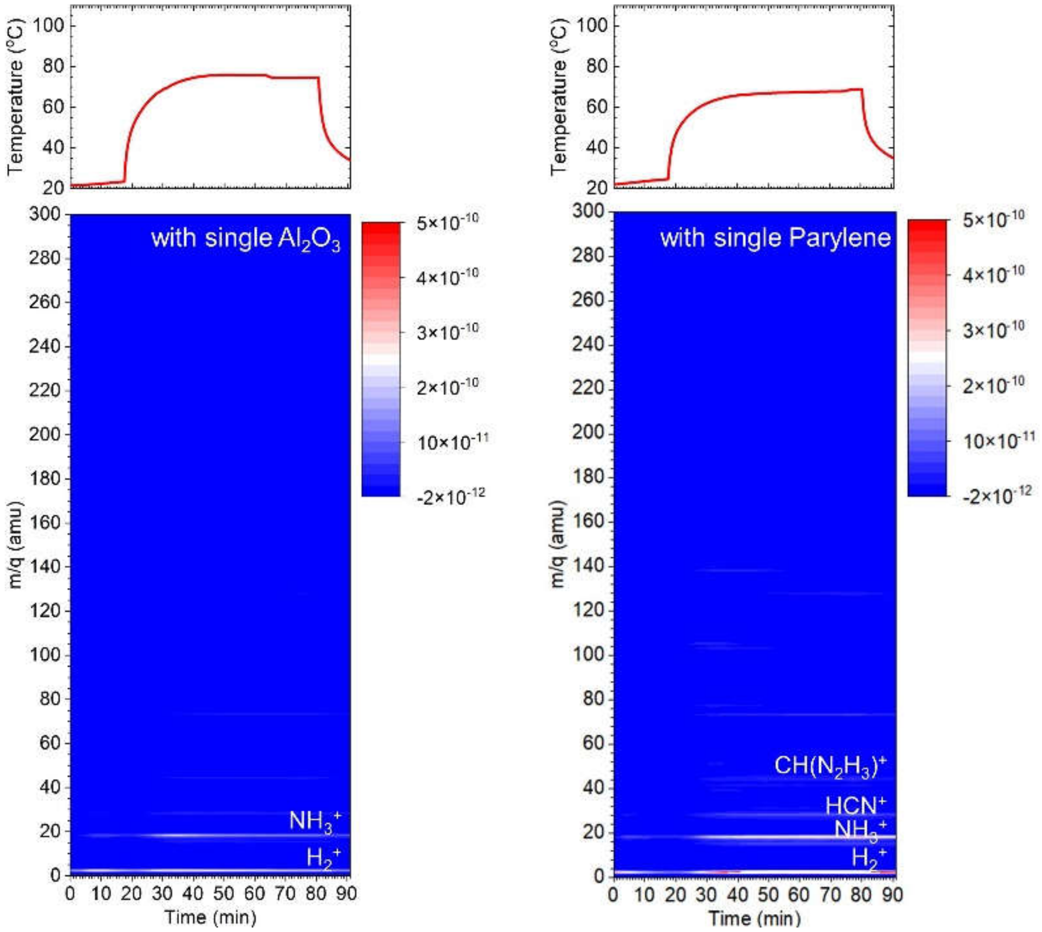

Supplementary Figure 9 | In situ illumination with temperature monitoring of mass spectrometry measurements of the perovskite films with single ALD-Al_2O_3 and single CVD-Parylene.

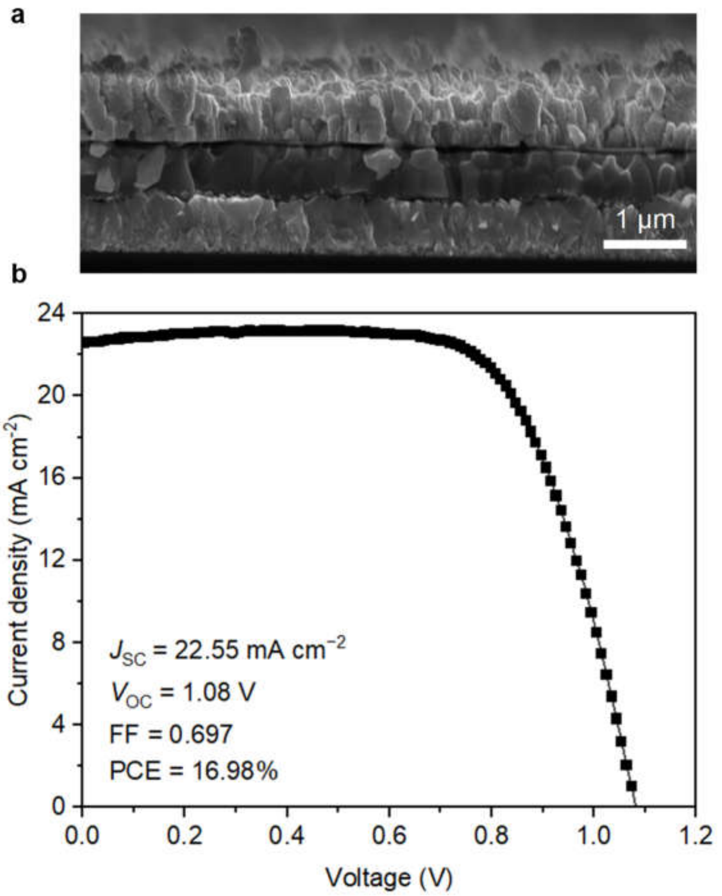

Supplementary Figure 10 | Cross-sectional morphology and corresponding *J-V* characteristic curves of perovskite solar cell devices with vapor-deposited Bi electrodes. a, Device SEM section diagram of the device with 1 μm T-Bi (FTO/NiMgLiO/PVSK//LiF/C_{60}/BCP/T-Bi). **b,** The corresponding *J-V* curve a typical small-area (1 cm^2) PSC with 1 μm T-Bi electrode based on a FACs absorber.

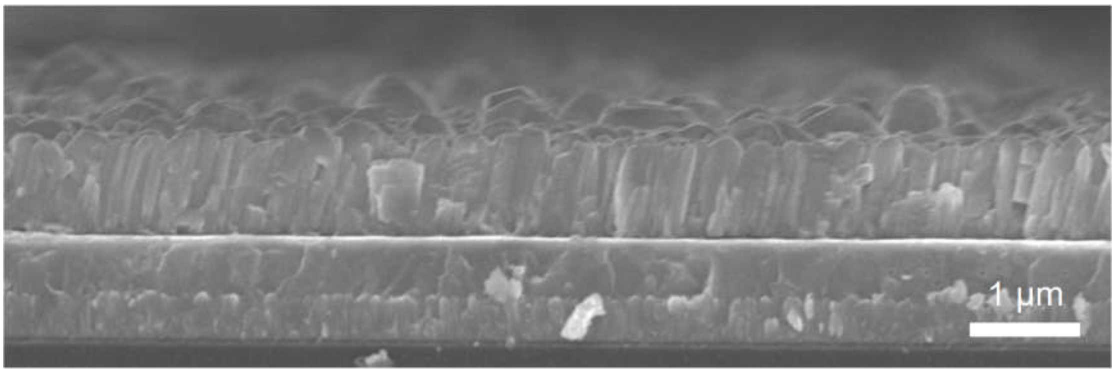

Supplementary Figure 11 | Cross-sectional SEM image of the device with 1 μm M-Bi (FTO/NiMgLiO/PVSK//LiF/C_{60}/BCP/M-Bi).

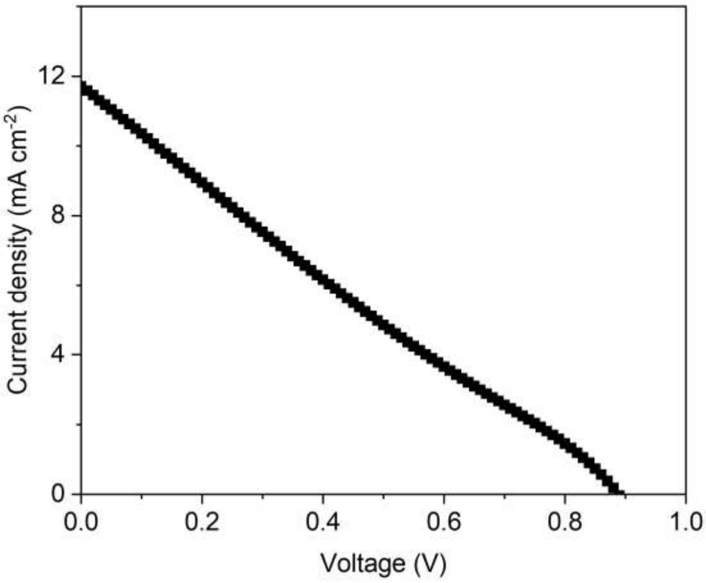

Supplementary Figure 12 | *J-V* curve of the device after sputtering Bi directly on the surface of the functional layer with the structure of FTO/NiMgLiO/PVSK/LiF/C_{60}/BCP/M-Bi.

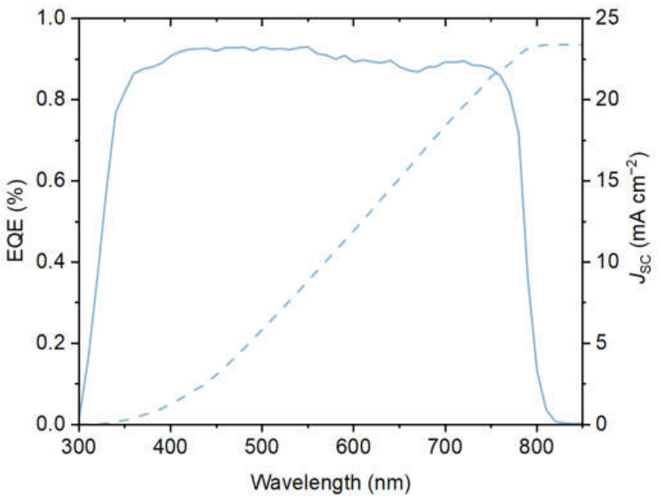

Supplementary Figure 13 | EQE spectra and integrated current density of the FTO/NiMgLiO/Perovskite/LiF/C_{60}/BCP/Bi device with an active area of 1 cm^2.

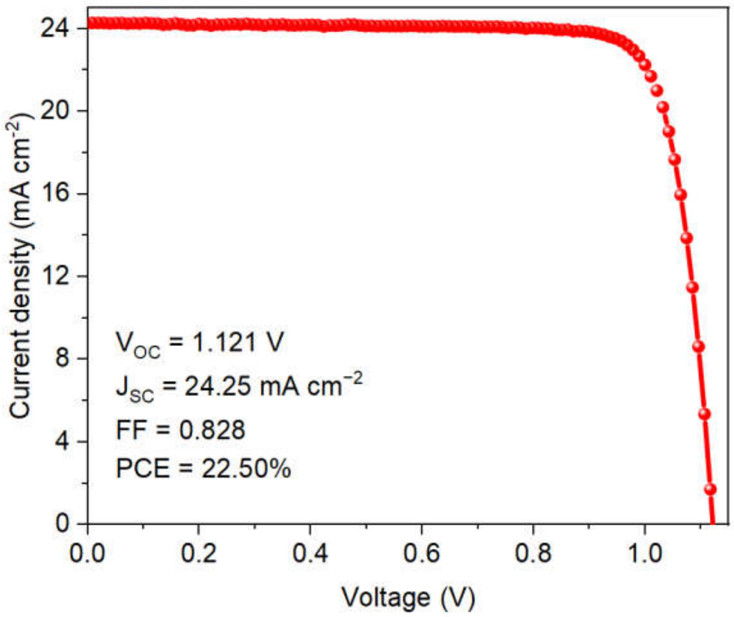

Supplementary Figure 14 | *J-V* curves of a typical small-area (0.09 cm^2) PSC with an 80 nm-thick Ag electrode based on FACs perovskite.

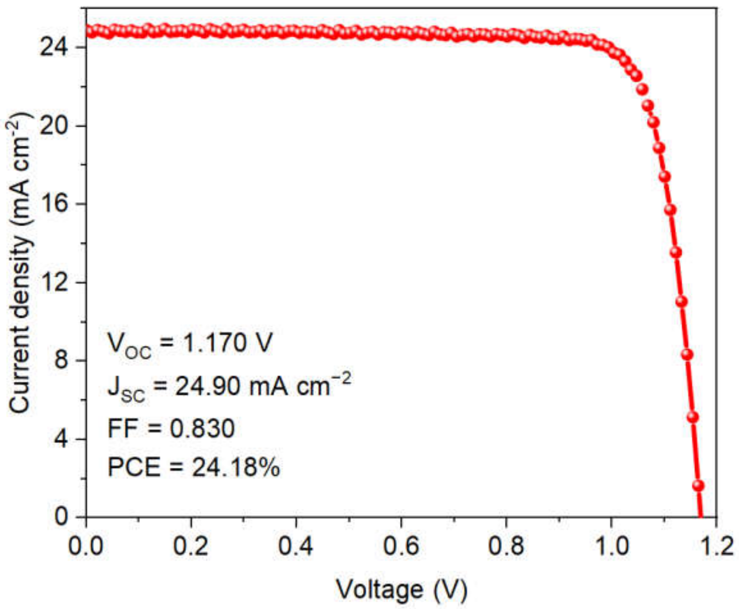

Supplementary Figure 15 | *J-V* curves of a typical small-area (0.09 cm^2) PSC with a device structure ITO/poly(triarylamine)(PTAA)/Perovskite/PEAI/PCBM/BCP/Ag.

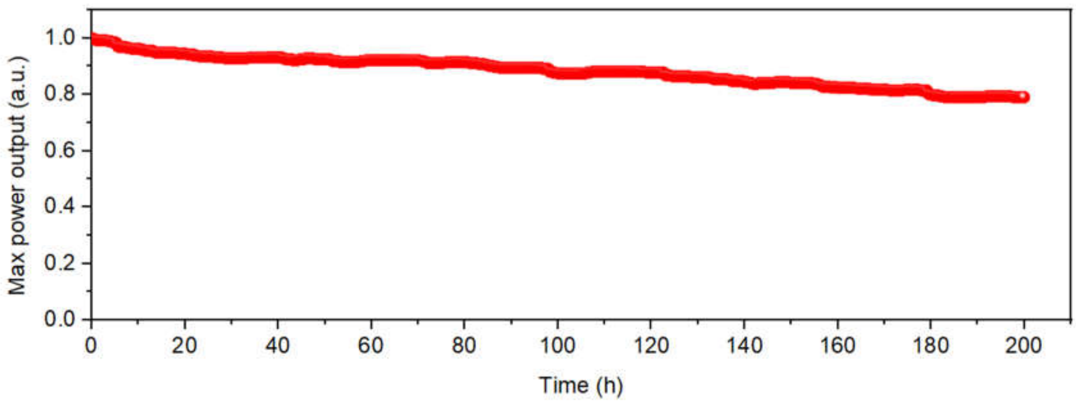

Supplementary Figure 16 | The maximum power output evolution of ITO/PTAA based device monitored by MPP tracking when aged at 75 °C under 1 sun equivalent white LED light illumination.

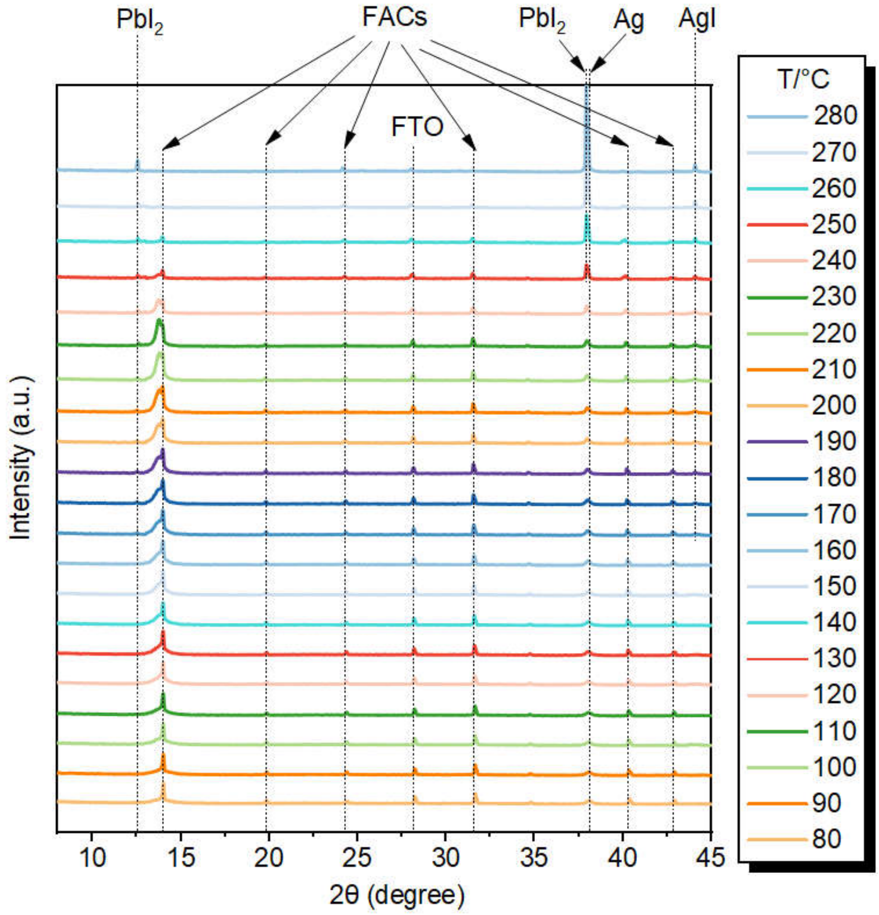

Supplementary Figure 17 | In situ heating XRD characterization of FACs perovskite/Ag/ALD-Al_2O_3/CVD-parylene sample.

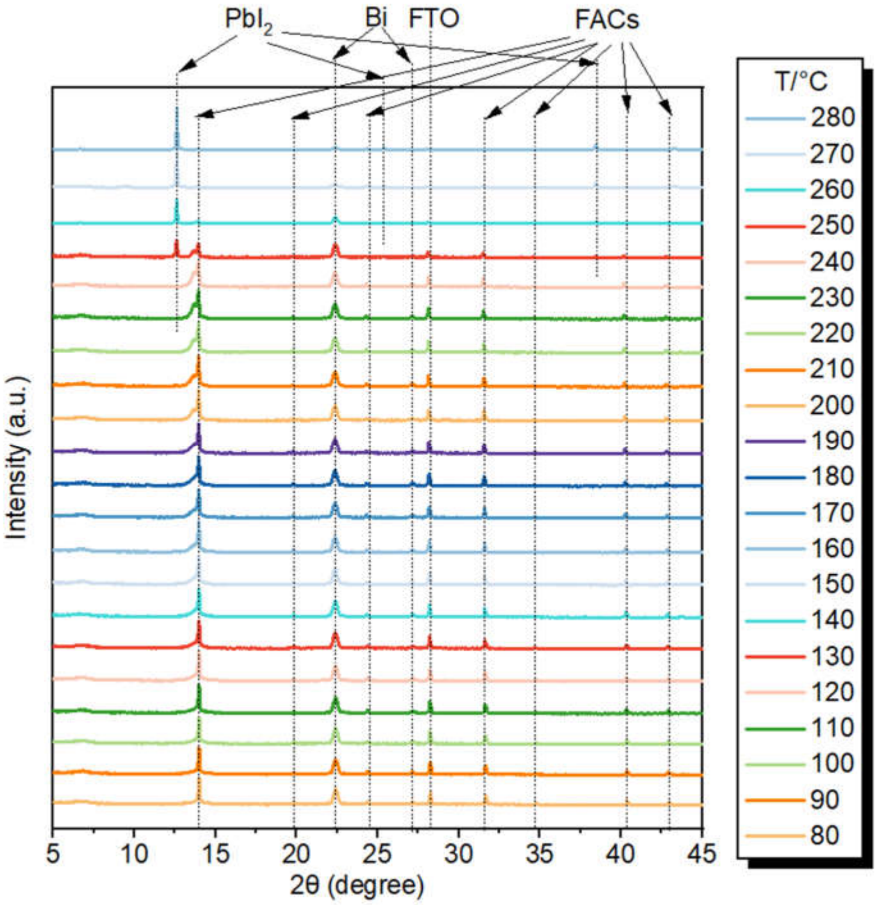

Supplementary Figure 18 | In situ heating XRD characterization of FACs perovskite/Bi/ALD-Al_2O_3/CVD-parylene sample.

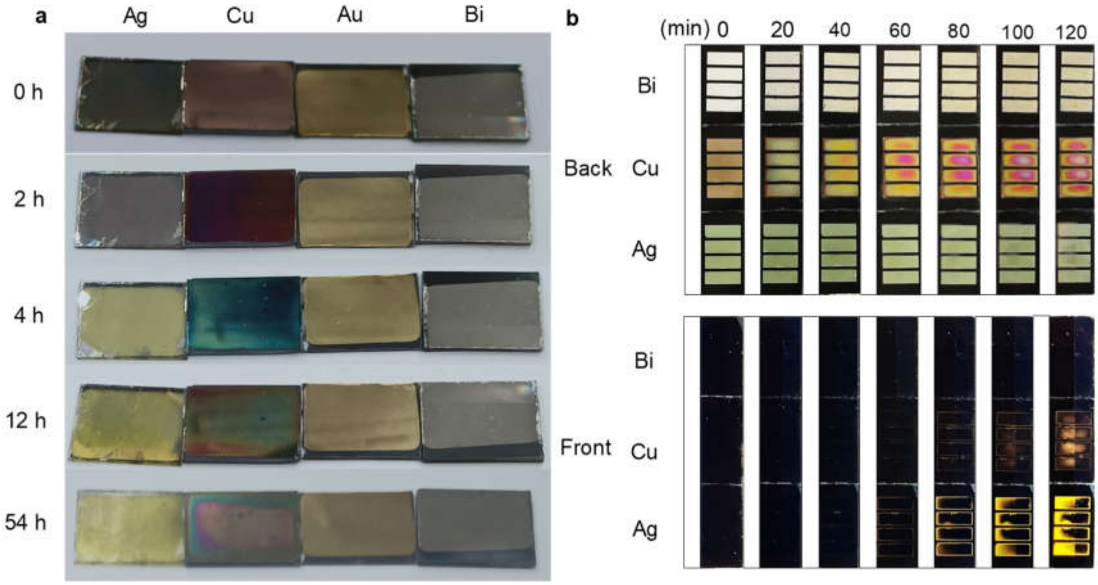

Supplementary Figure 19 | Induction of perovskite decomposition by metal corrosion. a, The optical photo evolution of the FACs perovskite samples with Ag, Cu, Au and Bi electrodes, respectively, under 1 sun equivalent white light LED soaking at 85 °C in N_2. **b,** The optical photos of thermal aging devices with Ag, Cu and Bi electrode. Aging condition: dark, ambient air and 175 °C, 120 min.

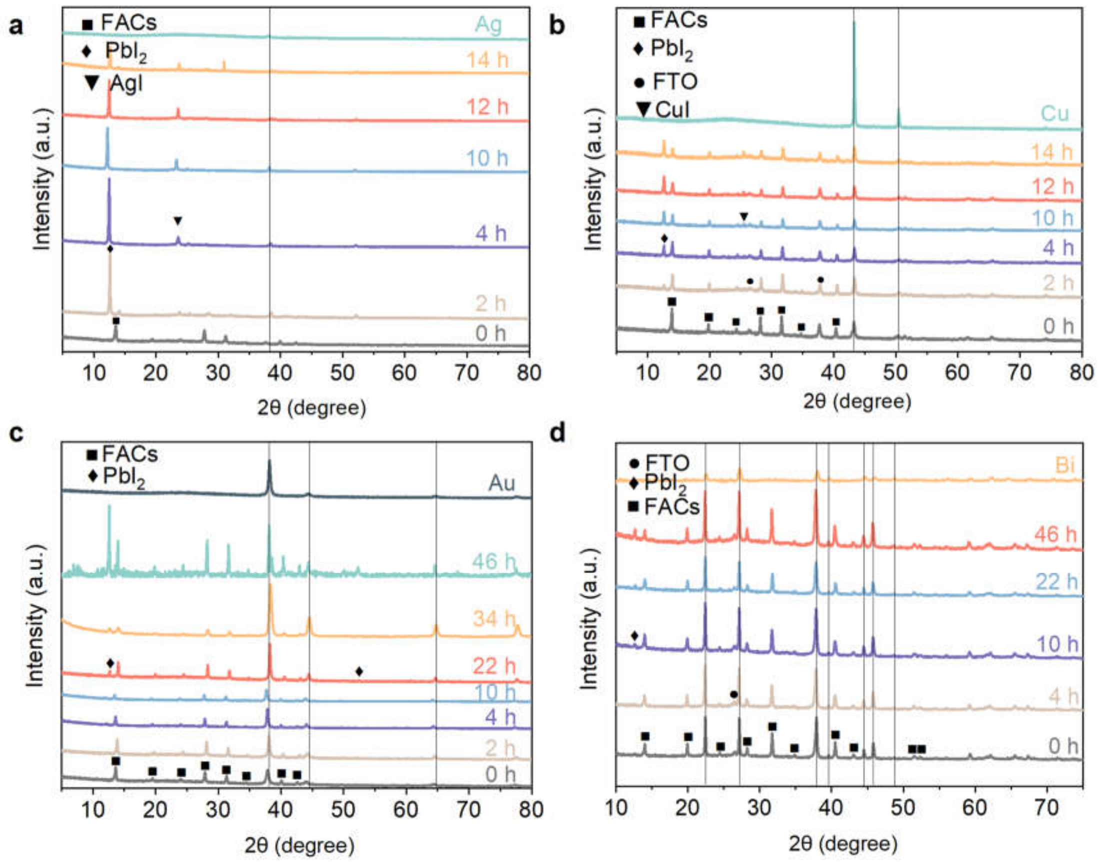

Supplementary Figure 20 | The XRD spectra evolution of the FACs films samples with Ag, Cu, Au and Bi electrode, respectively, under 1 sun equivalent white light LED soaking at 85 °C in N_2 atmosphere.

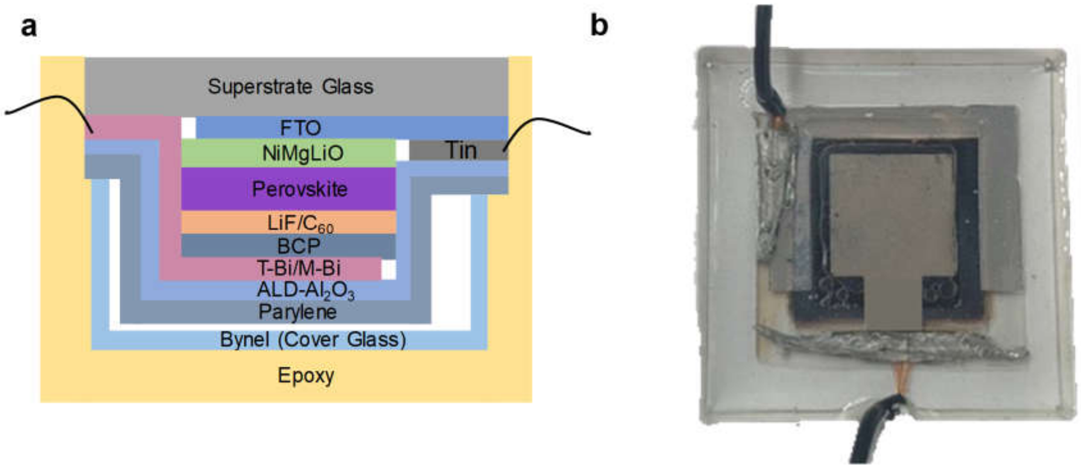

Supplementary Figure 21 | Illustration and physical demonstration of encapsulated perovskite solar cell devices. a, Schematic diagram of the device structure in this work: FTO/NiMgLiO/PVSK/LiF/C_{60}/BCP/Bi; the double barrier has strong compactness and hydrophobicity, preventing water or oxygen in the outside and perovskite decomposition on the inside; Bynel and epoxy are used to encapsulate furtherly (not to scale). **b,** Meatal-side view of PSC after packing.

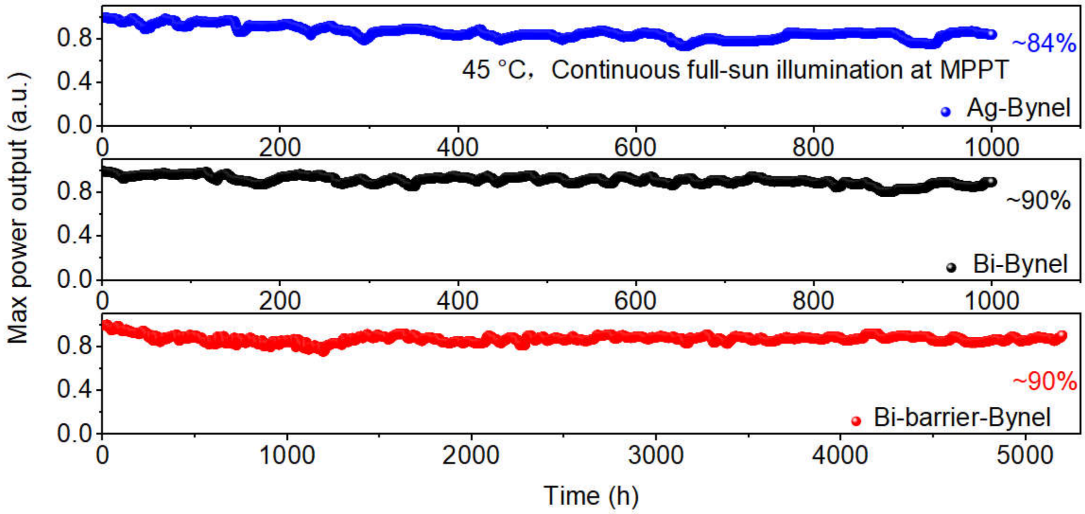

Supplementary Figure 22 | The maximum power output evolution of devices monitored by MPP tracking when aged at 45 °C under a white light LED illumination with the intensity of 1 sun equivalent.

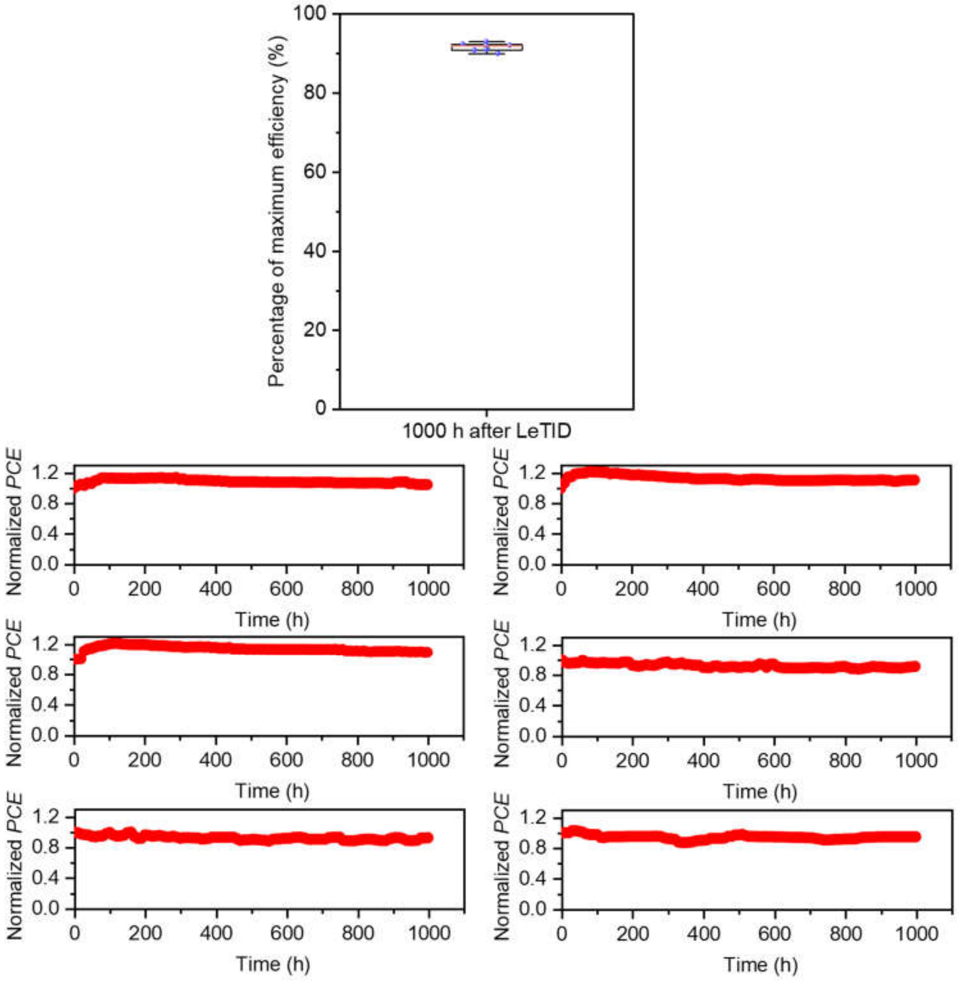

Supplementary Figure 23 | Statistical graph of the percentage of initial efficiency of the FTO/NiMgLiO/Perovskite/LiF/C_{60}/BCP/Bi/Al_2O_3/Parylene device with encapsulation after 1000 hours of aging under LeTID.

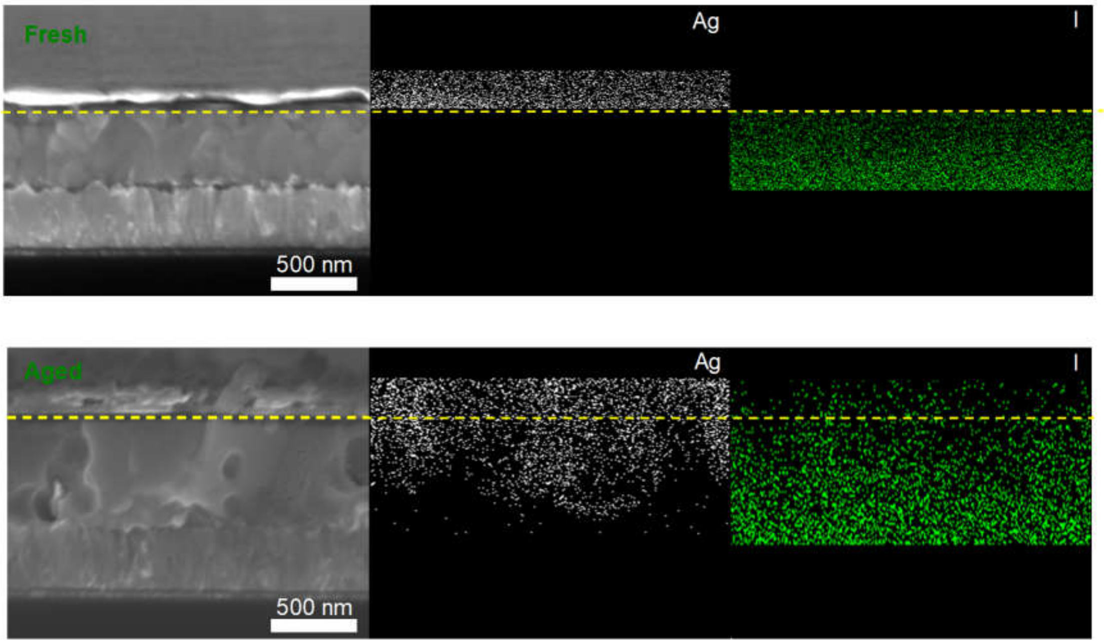

Supplementary Figure 24 | Cross-sectional SEM images and element distribution of FACs perovskite-based devices with a structure of FTO/NiMgLiO/perovskite/LiF/C_{60}/BCP/Ag before and after 1000 hours aging under operational conditions (aging condition: 1 sun equivalent white light LED soaking, 75°C, 1000 hours).

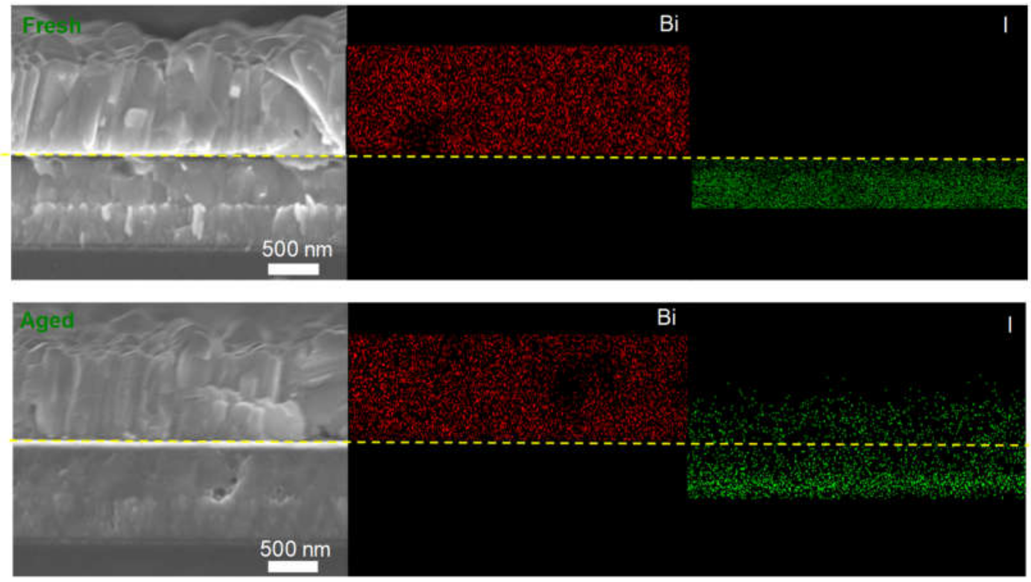

Supplementary Figure 25 | Cross-sectional SEM images and element distribution of FACs perovskite-based devices with a structure of FTO/NiMgLiO/perovskite/LiF/C_{60}/BCP/Bi before and after 1000 hours aging under operational conditions (aging condition: 1 sun equivalent white light LED soaking, 75°C, 1000 hours).

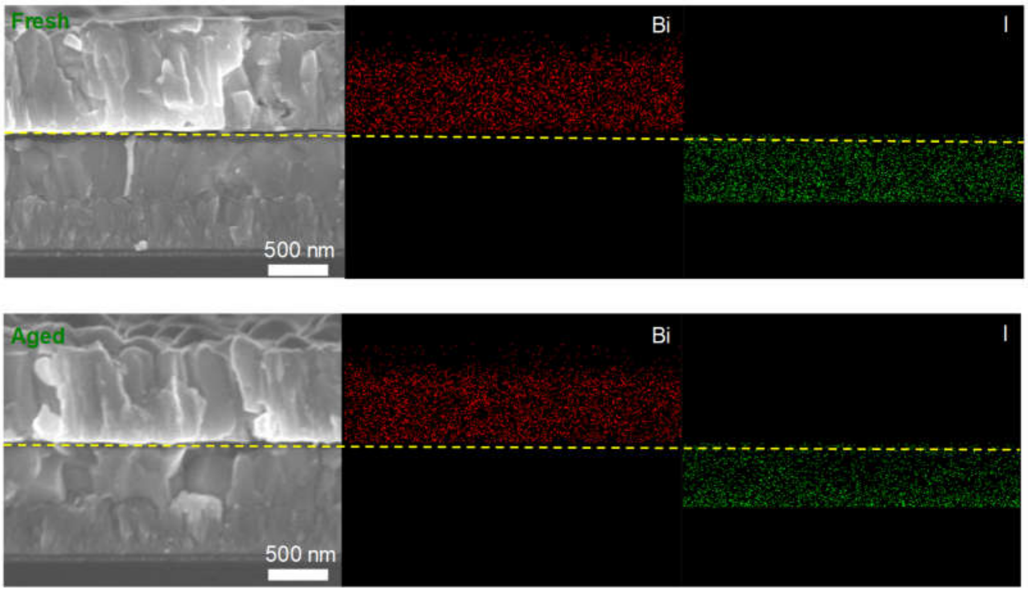

Supplementary Figure 26 | Cross-sectional SEM images and element distribution FACs perovskite-based devices with a structure of FTO/NiMgLiO/perovskite/LiF/C_{60}/BCP/Bi/ALD-Al_2O_3/CVD-Parylene before and after 1000 h aging under operational conditions (aging condition: 1 sun equivalent white light LED soaking ,75°C, 1000 hours).

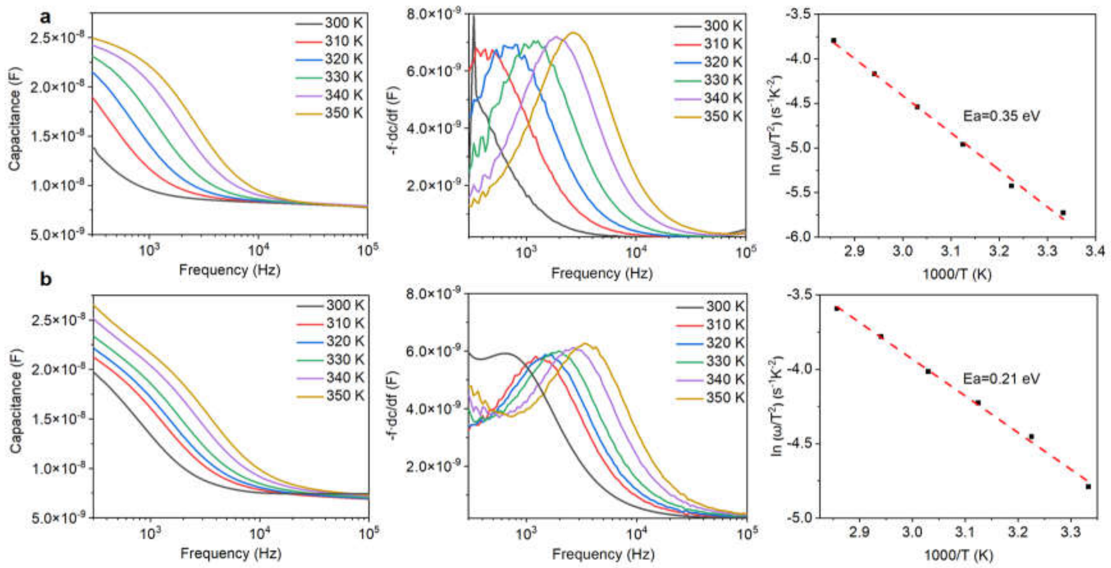

Supplementary Figure 27 | Capacitance-frequency (*C-f*) characterization results of the Ag-Bynel solar cell in the dark at selected temperatures (bias = 0 V) and Arrhenius plot of the characteristic frequencies to determine the defect activation energy before (**a**) and after (**b**) the long-term stability tests.

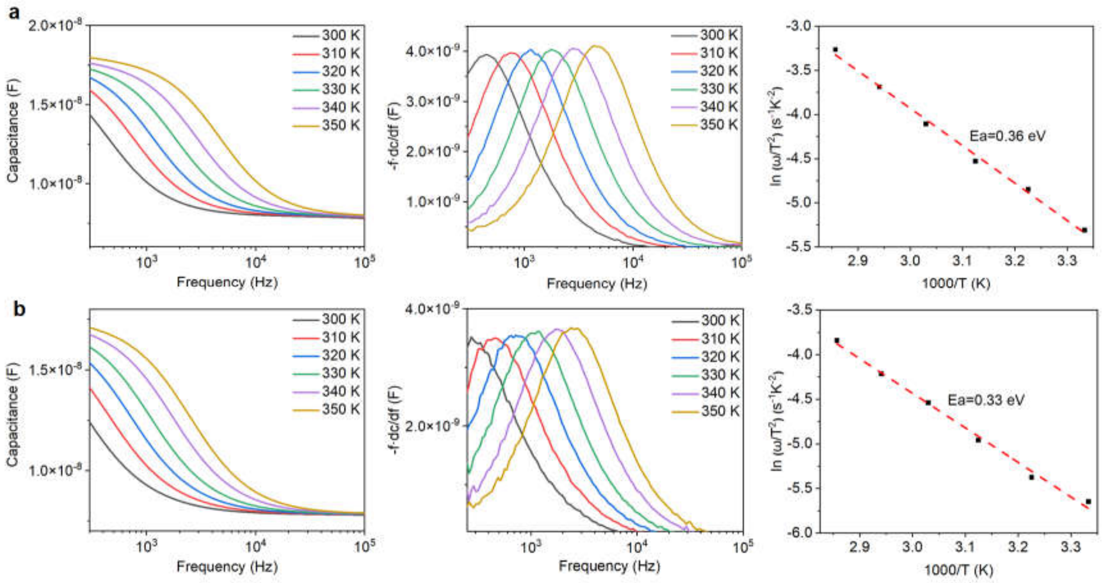

Supplementary Figure 28 | Capacitance-frequency (*C-f*) characterization results of the Bi- Barrier-Bynel solar cell in the dark at selected temperatures (bias = 0 V) and Arrhenius plot of the characteristic frequencies to determine the defect activation energy before (**a**) and after (**b**) the long-term stability tests.

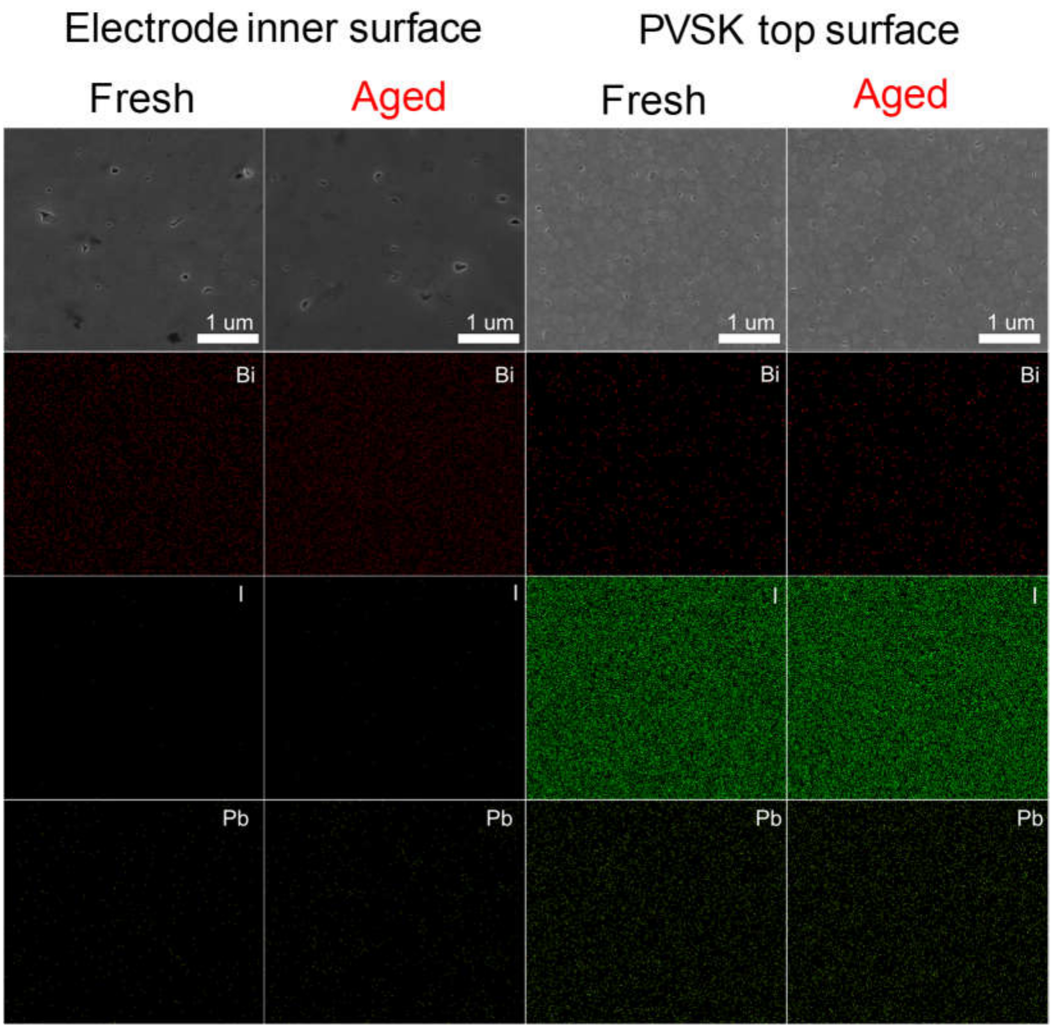

Supplementary Figure 29 | SEM images of the Bi electrodes' inner surfaces and perovskite top surfaces before and after aging under 1 sun equivalent white light LED soaking accompanied with thermal at 85°C for 100 hours in N_2 atmosphere. The structure of the samples is Glass/perovskite/C_{60}/Bi/ Al_2O_3/parylene. The samples were obtained by dissolving C_{60} in FACs-PSCs using chlorobenzene.

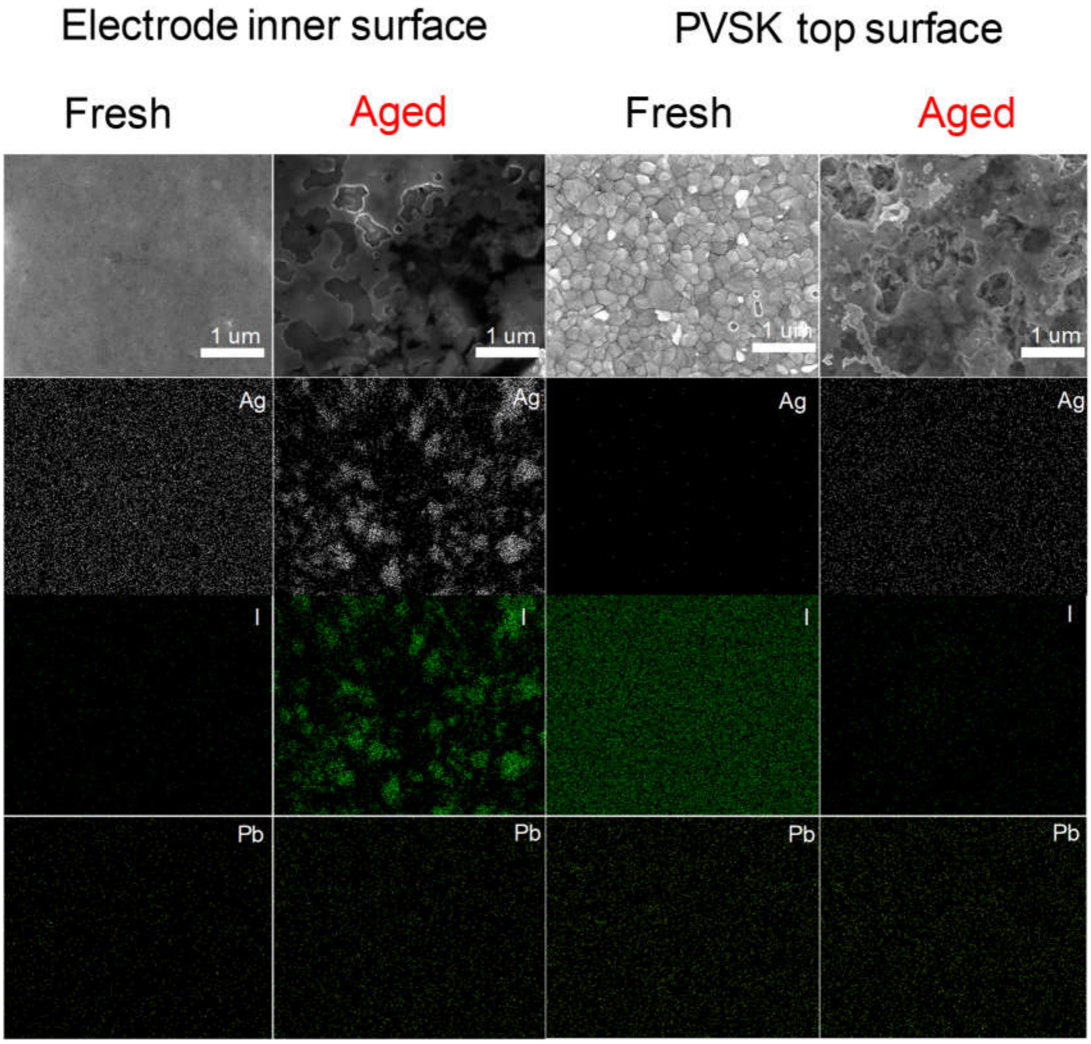

Supplementary Figure 30 | SEM images of the Ag electrodes' inner surfaces and perovskite top surfaces before and after aging under 1 sun equivalent white light LED soaking accompanied with thermal at 85°C for 100 hours in N_2 atmosphere. The structure of the samples is Glass/perovskite/C_{60}/Ag. The samples were obtained by dissolving C_{60} in FACs-PSCs using chlorobenzene.

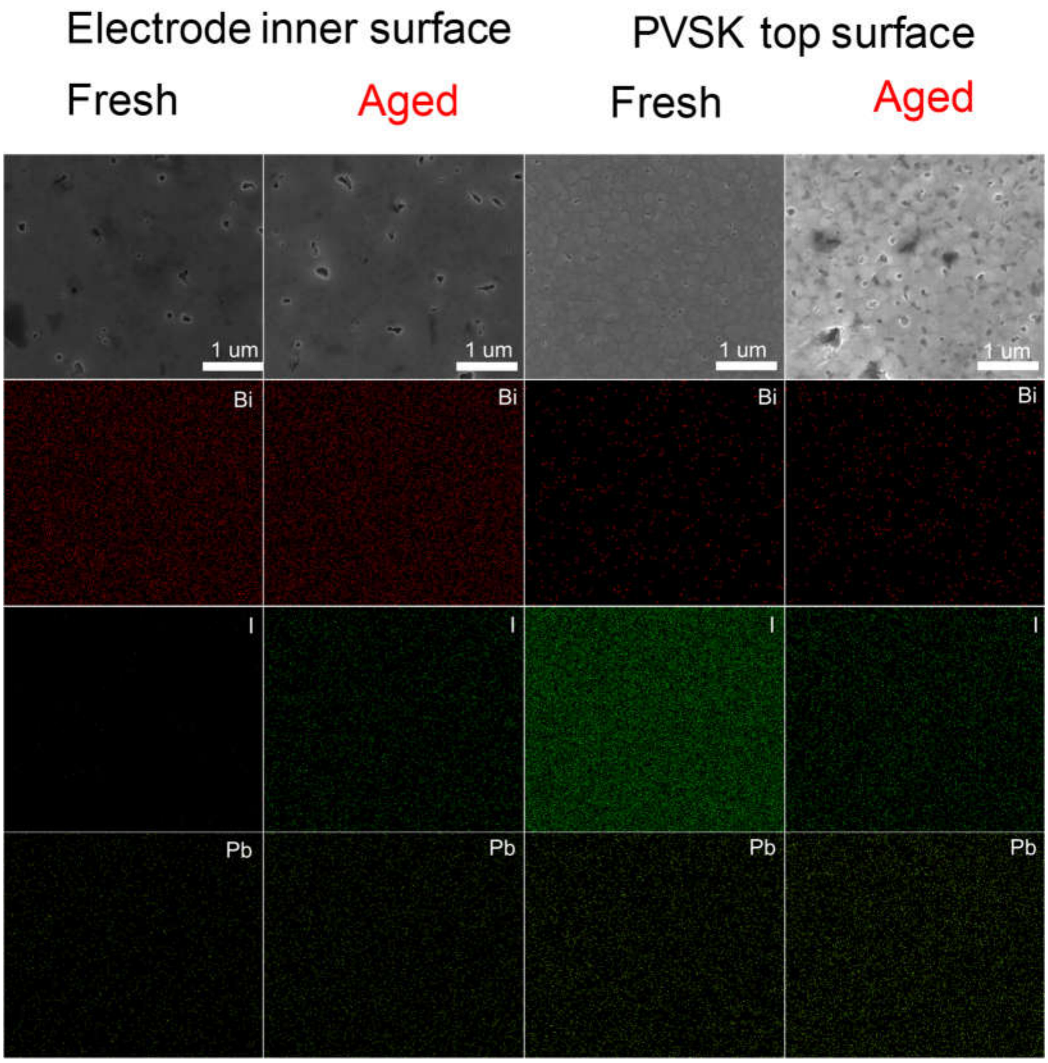

Supplementary Figure 31 | SEM images of the Bi electrodes' inner surfaces and perovskite top surfaces before and after aging under 1 sun equivalent white light LED soaking accompanied with thermal at 85°C for 100 hours in N_2 atmosphere. The structure of the samples is Glass/perovskite/C_{60}/Bi. The samples were obtained by dissolving C_{60} in FACs-PSCs using chlorobenzene.

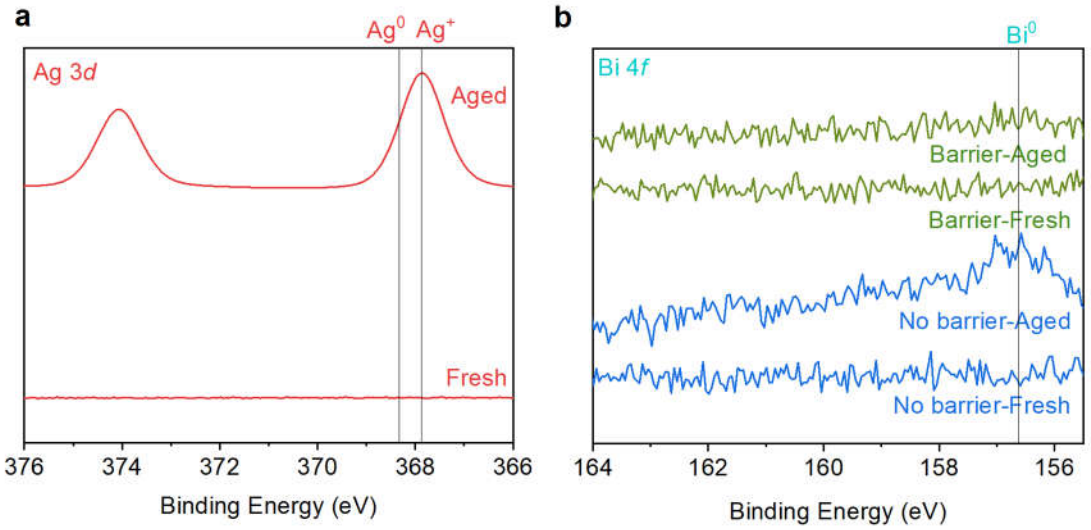

Supplementary Figure 32 | XPS spectra of Ag 3*d*, Bi 4*f* peak of the FACs films with the configuration of (a) Glass/PVSK/Ag, (b) Glass/PVSK/C_{60}/Bi (without barrier) and Glass/PVSK/C_{60}/Bi/ALD-Al_2O_3/Parylene (with barrier) before and after aging under 1 sun equivalent white light LED soaking accompanied with thermal at 85°C for 100 hours in N_2 atmosphere.

Supplementary Table 1 | The WVTR of different barriers.

Barrier	Al_2O_3	Parylene	Al_2O_3/Parylene
WVTR ($g^{-1}m^{-2}day^{-1}$)	2.24×10^{-4}	1.38×10^{-4}	7.12×10^{-5}

Supplementary Table 2 | The resistance of T-Bi varies with film thickness.

Film thickness (nm)	400	1200	1500
Sheet resistance (Ω square^{-1})	12.1	8.5	6.2

Supplementary Table 3 | Summary of PSCs devices' Long-time thermal (at ≥ 70 °C) and illumination stability with different encapsulation conditions. MPP is maximum at power point tracking stability under high temperature and light. "-" express there are no detailed statements in the source text.

Device structure	Initial PCE (%)	% of initial PCE after aging	Active area (cm^2)	Perovskite composition	Encapsulation method	Stability tests	Reference
FTO/NiMgLiO/PVSK/LiF/C_{60}/BCP/Bi	20.56	93	1	$FA_{0.85}Cs_{0.15}Pb(I_{0.95}Br_{0.05})_3$	ALD-Al_2O_3 with CVD-Parylene double layer, bynel with a cover glass	1 sun, MPP, 75 °C, 1000 h, ambient air without humidity control	This work
ITO/PEDOT:PSS/$FAPbI_{3-x}Br_x$/NDI-ID/Al	18.02	90	0.16	$CH(NH_2)_2PbI_{3-x}Br_x$	No	1 sun, 30% RH, 100 °C, 500 h	[4]
FTO/ALD-NiO/$Cs_{0.05}MA_{0.95}PbI_3$/PCBM/BCP/ALD-AZO/Ag	16.5	86.7	0.24	$Cs_{0.05}MA_{0.95}PbI_3$	-	1 sun, MPP, 85 °C, 500 h, ambient air	[5]
FTO/c-TiO_2/m-TiO_2/$FA_{0.83}MA_{0.17}Pb(I_{0.83}Br_{0.17})_3$/Spiro-OMeTAD/Cr/Au	13	83	0.26	$FA_{0.83}MA_{0.17}Pb(I_{0.83}Br_{0.17})_3$	No	1 sun, MPP, 75 °C, in N_2, 12 h	[6]
FTO/NiO/PVSK/PCBM/BCP/Cr/Cr_2O_3/Au	18	88	-	$(FA_{0.83}MA_{0.17})_{0.95}Cs_{0.05}Pb(I_{0.9}Br_{0.1})_3$	-	under continuous simulated full spectrum sunlight, 70-75 °C, 1800 h	[7]

FTO/PolyTPD:F4TCNQ/ $Cs_{0.17}FA_{0.83}Pb(I_{1-x}Br_x)_3$/PCBM/BCP/Cr/Au	14	85	0.0919	$Cs_{0.17}FA_{0.83}Pb(I_{0.77}Br_{0.23})_3$	-	under continuous simulated full spectrum sunlight, 85 °C, 1200 h	8
FTO/SnO_2/pero/ EH44/ MoOx/Al	18.5	94	0.059	$(FA_{0.79}MA_{0.16}Cs_{0.05})_{0.97}Pb(I_{0.84}Br_{0.16})_{2.97}$	No	Plasma lamp, resistive load, ~70 °C, RH 50%, air	9
FTO/c-TiO_2/mp-TiO_2/ pero/PTAA/Au	21.6	95	0.16	$(FA,MA,Cs,Rb)Pb(I,Br)_3$	-	White LED, MPP, 85 °C, N_2, 500 h	10
FTO/PTAA/HaP/ PCBM/AZO/Ag	19.32	68.72	1	$CH_3NH_3PbI_{3-x}Cl_x$	glass and UV-curable resins	under maximum power point condition (1 sun) at 85 °C	11
ITO/p-TPD/PFN/perovskite/SAM/C_{60}/Cu	16	100	-	$CsI_{0.05}[FA_{0.85}MA_{0.15}Pb(I_{0.85}Br_{0.15})_3]_{0.95}$	No	under maximum power point condition (1 sun) at 85 °C for 250 h in glovebox	12
FTO/NiO/PFDT @Perovskite/PCBM/BCP/PFDT @Ag	-	90.1	0.05	$Cs_{0.05}(FA_{0.85}MA_{0.15})_{0.95}Pb(I_{0.85}Br_{0.15})_3$	-	under maximum power point condition (1 sun) at 85 °C for 500 h in glovebox	13
ITO/PTAA/Perovskite/$PC_{61}BM$/Zr L_3:bis-C60/Ag	21	92	0.13	$Cs_{0.05}(FA_{0.85}MA_{0.15})_{0.95}Pb(I_{0.85}Br_{0.15})_3$	-	under maximum power point condition (1 sun)	14

						at 85 ℃ for 1000 h in glovebox	
FTO/SnO_x/SnO_2/Perovskite/HFDF-HTL/Au	23.04	92	0.0801	$(FAPbI_3)_{0.92}(MAPbBr_3)_{0.08}$	glass and UV-curable epoxy	under AM1.5G illumination (100 mW cm^{-2}) and heat stress at 85 °C	15
Glass/FTO/ZTO-ZnS/mp-TiO_2/perovskite/CuI/Carbon	15.9	85	8	$Cs_{0.05}(FA_{0.85}MA_{0.15})_{0.95}Pb(Br_{0.15}I_{0.85})_3$	-	after 1000 h at 85 °C in air with a relative humidity of 85% under maximum power point and continuous AM 1.5G illumination	16
ITO/NiO/Perovskite/PCBM+C60/BCP/Cr/Au	22	91	0.1	CsFAMAPbIBr	a cover glass and UV adhesive (LT-U001, Lumtec)	under one-sun illumination at 75 °C for 1000 h	17
FTO/$ZnTiO_3$/FACsPbI_3/PVT/Spiro-OMeTAD/PVT/Au	-	90.57	18	FACsPbI_3	a hot-melt butyl sealant	85 °C and 60% RH for 300 h under 1 sun	18

Supplementary References

1. Lu, Q. *et al.* A review on encapsulation technology from organic light emitting diodes to organic and perovskite solar cells. *Adv. Funct. Mater.* **31**, 2100151 (2021).
2. Van Hemmen, J. *et al.* Plasma and thermal ALD of Al_2O_3 in a commercial 200 mm ALD reactor. *J. Electrochem. Soc.* **154**, G165 (2007).
3. Jakschik, S. *et al.* Crystallization behavior of thin ALD-Al_2O_3 films. *Thin Solid Films* **425**, 216-220 (2003).
4. Jung, S.-K. *et al.* Nonfullerene electron transporting material based on naphthalene diimide small molecule for highly stable perovskite solar cells with efficiency exceeding 20%. *Adv. Funct. Mater.* **28**, 1800346 (2018).
5. Seo, S., Jeong, S., Bae, C., Park, N.-G. & Shin, H. Perovskite solar cells with inorganic electron- and hole-transport layers exhibiting long-term (≈500 h) stability at 85 °C under continuous 1 sun illumination in ambient air. *Adv. Mater.* **30**, 1801010 (2018).
6. Domanski, K. *et al.* Not all that glitters is gold: metal-migration-induced degradation in perovskite solar cells. *ACS Nano* **10**, 6306-6314 (2016).
7. Bai, S. *et al.* Planar perovskite solar cells with long-term stability using ionic liquid additives. *Nature* **571**, 245-250 (2019).
8. Lin, Y.-H. *et al.* A piperidinium salt stabilizes efficient metal-halide perovskite solar cells. *Science* **369**, 96-102 (2020).
9. Christians, J. A. *et al.* Tailored interfaces of unencapsulated perovskite solar cells for >1,000 hour operational stability. *Nat. Energy* **3**, 68-74 (2018).
10. Saliba, M. *et al.* Incorporation of rubidium cations into perovskite solar cells improves photovoltaic performance. *Science* **354**, 206-209 (2016).
11. Khadka, D. B., Shirai, Y., Yanagida, M. & Miyano, K. Insights into accelerated degradation of perovskite solar cells under continuous illumination driven by thermal stress and interfacial junction. *ACS Appl. Energy Mater.* **4**, 11121-11132 (2021).
12. Wolff, C. M. *et al.* Perfluorinated self-assembled monolayers enhance the stability and efficiency of inverted perovskite solar cells. *ACS Nano* **14**, 1445-1456 (2020).
13. Zhang, H. *et al.* Design of superhydrophobic surfaces for stable perovskite solar cells with reducing lead leakage. *Adv. Energy Mater.* **11**, 2102281 (2021).
14. Wu, S. *et al.* 2D metal–organic framework for stable perovskite solar cells with minimized lead leakage. *Nat. Nanotechnol.* **15**, 934-940 (2020).
15. Wang, T. *et al.* Transporting holes stably under iodide invasion in efficient perovskite solar cells. *Science* **377**, 1227-1232 (2022).
16. Han, F. *et al.* Hyperstable perovskite solar cells without ion migration and metal diffusion based on ZnS segregated cubic $ZnTiO_3$ electron transport layers. *Sol. RRL* **5**, 2000654 (2021).
17. Cao, Q. *et al.* Efficient and stable inverted perovskite solar cells with very high fill factors via incorporation of star-shaped polymer. *Sci. Adv.* **7**, eabg0633 (2021).
18. Feng, Q. *et al.* Governing PbI_6 octahedral frameworks for high-stability perovskite solar modules. *Energy Environ. Sci.* **15**, 4404-4413 (2022).
