# Supplementary material for: Modulation of perovskite degradation with multiple-barrier for light-heat stable perovskite solar cells
Source: Nat Commun. 2023 Sep 30;14:6120. doi: 10.1038/s41467-023-41856-9 (PMC10542753; doi:10.1038/s41467-023-41856-9)
Supplement: Supplementary file 3 — Description of Additional Supplementary Files [file 41467_2023_41856_MOESM3_ESM.pdf]

File name: Supplementary Movie 1
Description: The water-soaking test of the perovskite films with different encapsulation conditions.

File name: Supplementary Movie 2
Description: The equipment for in situ mass spectrometry measurements.

File name: Supplementary Movie 3
Description: A water immersing test of a large-area perovskite film (16×13 cm^2) with ALD-Al_2O_3/CVD-parylene barrier.
