# Supplementary material for: Modulation of perovskite degradation with multiple-barrier for light-heat stable perovskite solar cells
Source: Nat Commun. 2023 Sep 30;14:6120. doi: 10.1038/s41467-023-41856-9 (PMC10542753; doi:10.1038/s41467-023-41856-9)
Supplement: Supplementary file 7 — Reporting Summary [file 41467_2023_41856_MOESM7_ESM.pdf]

nature portfolio

Corresponding author(s): Wei Chen, Yabing Qi, Zonghao Liu

Solar Cells Reporting Summary

Nature Portfolio wishes to improve the reproducibility of the work that we publish. This form is intended for publication with all accepted papers reporting the characterization of photovoltaic devices and provides structure for consistency and transparency in reporting. Some list items might not apply to an individual manuscript, but all fields must be completed for clarity.

For further information on Nature Research policies, including our data availability policy, see Authors & Referees.

Experimental design

Please check the following details are reported in the manuscript, and provide a brief description or explanation where applicable.

1. Dimensions

Item	Yes/No	Description
Area of the tested solar cells	☒ Yes	Page 5 in the manuscript.
	☐ No	*Explain why this information is not reported/not relevant.*
Method used to determine the device area	☐ Yes	*Provide a description of the method and state where this information can be found in the text.*
	☒ No	The area of 0.09 cm2 and 1 cm2 are determined by the designed shadow mask and easy to be measured by a caliper.

2. Current-voltage characterization

Item	Yes/No	Description
Current density-voltage (J-V) plots in both forward and backward direction	☒ Yes	Figure 3 in the manuscript.
	☐ No	
Voltage scan conditions	☒ Yes	Page 8 in the manuscript
	☐ No	*Explain why this information is not reported/not relevant.*
Test environment	☒ Yes	At each test instruction section in the manuscript.
	☐ No	*Explain why this information is not reported/not relevant.*
Protocol for preconditioning of the device before its characterization	☒ Yes	At each test instruction section in the manuscript.
	☐ No	*Explain why this information is not reported/not relevant.*
Stability of the J-V characteristic	☒ Yes	Figure 3 in the manuscript
	☐ No	*Explain why this information is not reported/not relevant.*

3. Hysteresis or any other unusual behaviour

Item	Yes/No	Description
Description of the unusual behaviour observed during the characterization	☐ Yes	*Provide a description of hysteresis or any other unusual behaviour observed during the characterization.*
	☒ No	There is no serious hysteresis in our devices.
Related experimental data	☒ Yes	Figure 3 in the manuscrip
	☐ No	*Explain why this information is not reported/not relevant.*

4. Efficiency

Item	Yes/No	Description
External quantum efficiency (EQE) or incident photons to current efficiency (IPCE)	☒ Yes	Supplementary Figure 13
	☐ No	*Explain why this information is not reported/not relevant.*
A comparison between the integrated response under the standard reference spectrum and the response measure under the simulator	☒ Yes	The integrated Jsc values are consistent with those from J-V measurements.
	☐ No	*Explain why this information is not reported/not relevant.*

For tandem solar cells, the bias illumination and bias voltage used for each subcell

☐ Yes
☒ No

Provide a description of the measurement conditions.

Not tandem solar cells

5. Calibration

Light source and reference cell or sensor used for the characterization

☒ Yes
☐ No

In the characterization and measurement section of the manuscript.

Explain why this information is not reported/not relevant.

Confirmation that the reference cell was calibrated and certified

☒ Yes
☐ No

In the characterization and measurement section of the manuscript.

Explain why this information is not reported/not relevant.

Calculation of spectral mismatch between the reference cell and the devices under test

☒ Yes
☐ No

The light spectrum used for measurements matches well with the reference cell and AM1.5, and we used mismatched factor of 1 for all devices.

Explain why this information is not reported/not relevant.

6. Mask/aperture

Size of the mask/aperture used during testing

☒ Yes
☐ No

At the device performance description section.

Explain why this information is not reported/not relevant.

Variation of the measured short-circuit current density with the mask/aperture area

☐ Yes
☒ No

Report the difference in the short-circuit current density values measured with the mask and aperture area.

All J-V curves were measured with mask.

7. Performance certification

Identity of the independent certification laboratory that confirmed the photovoltaic performance

☐ Yes
☒ No

Identify the independent certification laboratory.

1. the light source of simulated AM 1.5G is calibrated by a certified silicon solar cell
2.the device are almost hysteresis-free
3.the focus of this work is the light-heat (LeTID) stability

A copy of any certificate(s)

☐ Yes
☒ No

Certificate copies should be provided in the Supplementary information. Please state the supplementary item number.

not applicable

8. Statistics

Number of solar cells tested

☒ Yes
☐ No

Supplementary Figure 2 and Supplementary Figure 23

Explain why this information is not reported/not relevant.

Statistical analysis of the device performance

☒ Yes
☐ No

Page 5 and Page 11

Explain why this information is not reported/not relevant.

9. Long-term stability analysis

Type of analysis, bias conditions and environmental conditions

☒ Yes
☐ No

Fig.5 in the manuscript and Supplementary Figure 22 in the supporting information.

Explain why this information is not reported/not relevant.
